# DNA nanorobot for mitochondria-targeted microRNAs detection and tailored regulation

**DOI:** 10.7150/thno.105762

**Published:** 2025-03-21

**Authors:** Ping Huang, Xiaoqi Tang, Shuang Zhao, Jie Luo, Yu Tang, Binpan Wang, Zuowei Xie, Xianlan Wu, Shuang Xie, Ming Chen, Kai Chang

**Affiliations:** 1 Department of Clinical Laboratory Medicine, Southwest Hospital, Third Military Medical University (Army Medical University), 30 Gaotanyan, Shapingba District, Chongqing 400038, P. R. China.; 2 College of Pharmacy and Laboratory Medicine, Third Military Medical University (Army Medical University), 30 Gaotanyan, Shapingba District, Chongqing 400038, P. R. China.

**Keywords:** DNA nanorobot, mitochondrial microRNA, doxorubicin, catalytic hairpin assembly, mitochondria

## Abstract

**Rationale:** Mitochondrial miRNAs (mitomiRs) are crucial regulators of mitochondrial functions and play pivotal roles in tumorigenesis and cancer progression. Nevertheless, direct monitoring mitomiRs and regulating mitochondrial function at the subcellular level remains challenging.

**Methods:** In this study, we present a versatile DNA framework-based nanorobot for synchronous ultrasensitive detection of mitochondrial miRNAs (mitomiRs) and modulation of the mitochondria-associated apoptosis process. The DNA nanorobot features a tetrahedral nucleic acid framework as its structural body, two DNA hairpins (H1 and H2) as functional arms, and a mitochondria-targeting triphenylphosphine (TPP) group as the command center. The DNA nanorobot was comprehensively characterized for its morphological properties, mitochondria-targeting capacity, mitomiRs detection performance, DOX-loading and release behaviors, and antineoplastic effects both *in vitro and in vivo*.

**Results:** Upon recognizing mitomiRs, the arms of the DNA nanorobot activate and trigger spatially restricted catalytic hairpin assembly (CHA) reactions with accelerated kinetics to generate amplified fluorescence signals. Additionally, the lipophilic anticancer drug doxorubicin (DOX) encapsulated within the DNA nanorobot induces reactive oxygen species (ROS) production, leading to mitochondria damage and promoting mitochondria-associated apoptosis in tumor cells.

**Conclusion:** This newly developed DNA nanorobot provides a multifunctional platform for precise mitochondria-targeted diagnosis and enhanced therapeutic efficacy, advancing innovative strategies for mitochondria-focused tumor diagnosis and treatment.

## Introduction

Mitochondria play a pivotal role in regulating cellular activities and cell death 1. Mitochondrial miRNAs (mitomiRs), a unique class of miRNAs localized in mitochondria and cytoplasm, regulate mitochondrial gene expression and function 2. Dysregulated mitochondrial function, often modulated by abnormal mitomiRs activity, is associated with various diseases including metabolic disorders, aging, and cancer 3. Analyzing the spatiotemporal expression of mitomiRs in living cells provides valuable insights into the mitochondrial function and cellular states. Current advancements in intracellular imaging nanoprobes and organelle-targeting technologies have aimed at elucidating the dynamic distribution and expression of mitomiRs 4, 5. However, these probes are primarily designed for *in situ* imaging and have not yet been utilized to synergistically regulate mitochondrial function.

DNA nanorobots represent an emerging nanotechnology for constructing intelligent biosensors 6 and drug carriers 7. Nanorobots with diverse morphological characteristics can be optimally engineered to perform customized functions by precise DNA sequence design 8-10. Tetrahedral framework nucleic acids (tFNA) stand out as a favorable structural framework for building DNA nanorobots due to their controllable size, tailorable functionality, and precise addressability 11. The tFNA-based DNA nanorobots are suited for intracellular miRNA analysis because their three-dimensional configuration facilitates endocytosis and enhances nuclease resistance 12. Moreover, the structural property of tFNA provides multiple editable sites, which can be used for the integration of targeting 13, detection 14, and therapeutic 15 modules into the nanorobots. As a result, tFNA-based structures offer a robust foundation for developing nanorobots capable of simultaneous intracellular detection and regulation.

Catalytic hairpin assembly (CHA) is extensively adapted to diverse reaction systems and the detection of multifarious biomarkers 16. Its enzyme-free, high-efficiency, and isothermal amplification properties make it a popular choice for constructing DNA nanomachines 14. Doxorubicin (Dox) is currently the primary antineoplastic drug in clinical treatment owing to its pleiotropic activity. 17. However, its application is limited by dose-dependent cardiotoxicity. Researchers are developing nanocarriers that endow Dox with tumor-targeting capabilities to mitigate this issue. Pre-linking Dox at the 5′ or 3′ ends of single-strand DNA or inserting it in the DNA double helices via physical conjugate 18 can integrate therapeutic modules into DNA nanomachines. Triphenylphosphonium (TPP) 19, a cationic for targeting mitochondria, facilitates the navigation of nanodevices to mitochondria via electrostatic interaction. Based on this, researchers have developed near-infrared (NIR) light-controlled 20, pH-responsive 21, and logic operation-controlled 22 nanodevices for mitomiRs sensing. Furthermore, Sun et al. proposed a dual-targeting DNA nanomachine for antitumor treatment, ensuring precise delivery of chemotherapeutic drugs to mitochondria in cancer cells 23. These advancements lay the groundwork for constructing multifunctional nanorobots capable of mitochondrial biomarker analysis and functional regulation.

**Scheme 1:**
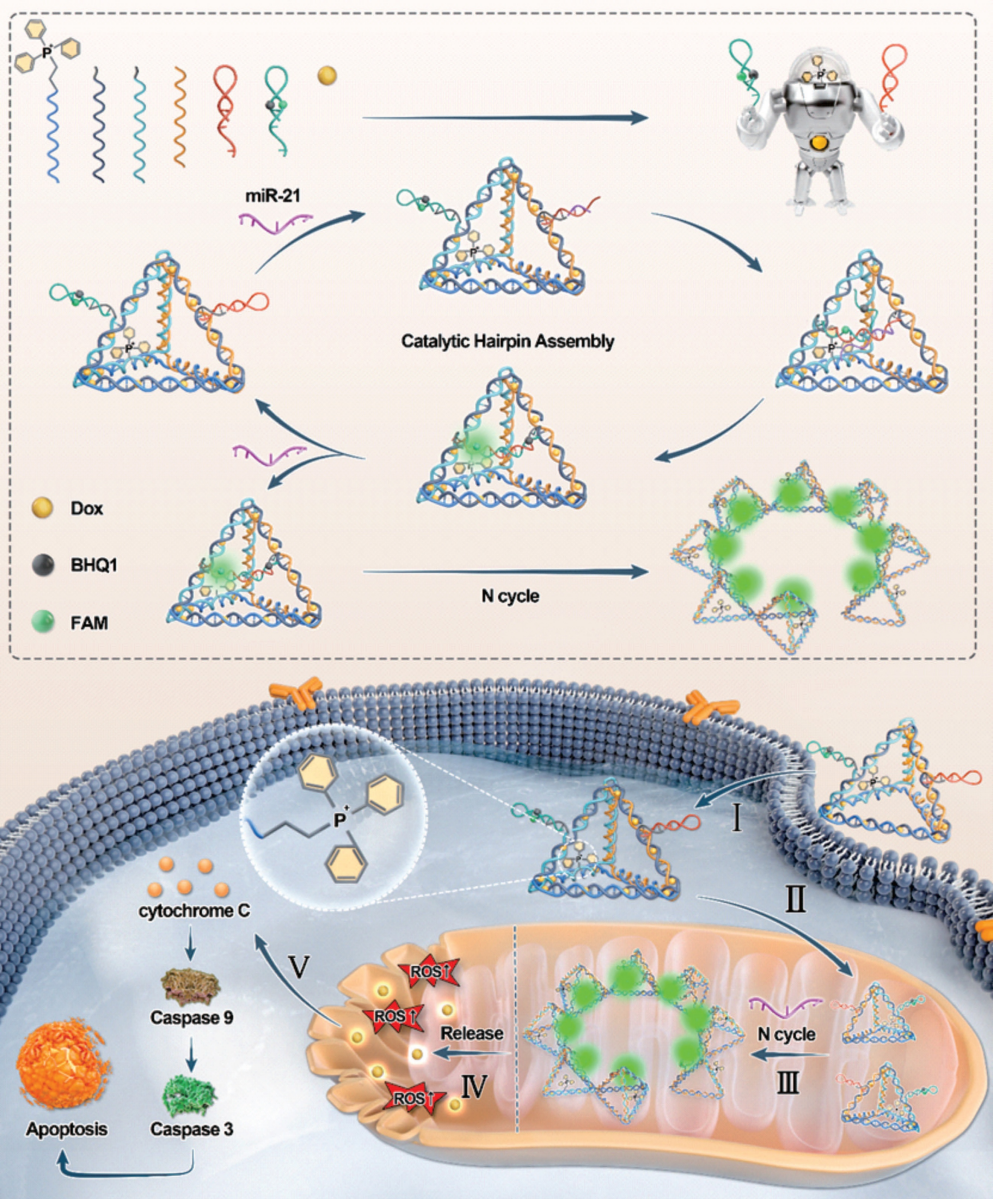
The upper panel illustrates a schematic of the MTDN self-assembly process, while the lower panel depicts the mechanism of mitochondrial miRNA detection and mitochondrial function regulation by MTDN.

In this study, we developed a multifunctional DNA nanorobot designed to target mitochondria for spatially imaging mitomiRs and inducing mitochondria-mediated apoptosis (Scheme 1). The nanorobot's command center was formed by linking an amino-modified single-stranded DNA (ssDNA) with triphenylphosphonium (TPP). The remaining portion of this ssDNA was hybridized with other ssDNAs to assemble a three-dimensional robotic torso through base pairing, which served as a carrier for Dox loading. In this way, TPP is scarcely restricted in the interior of the tetrahedral framework, ensuring its mitochondrial targeting capability. Nanorobotic arms were subsequently grafted onto the torso at a constant temperature, completing the assembly of mitochondria-targeting DNA nanorobots (MTDN). The MTDN provides a novel approach for the specific, sensitive, and rapid monitoring of dynamic mitomiRs levels and the synchronous activation of the mitochondria damage-induced antitumor therapy.

## Results

### Assembly of MTDN

**Figure 1:**
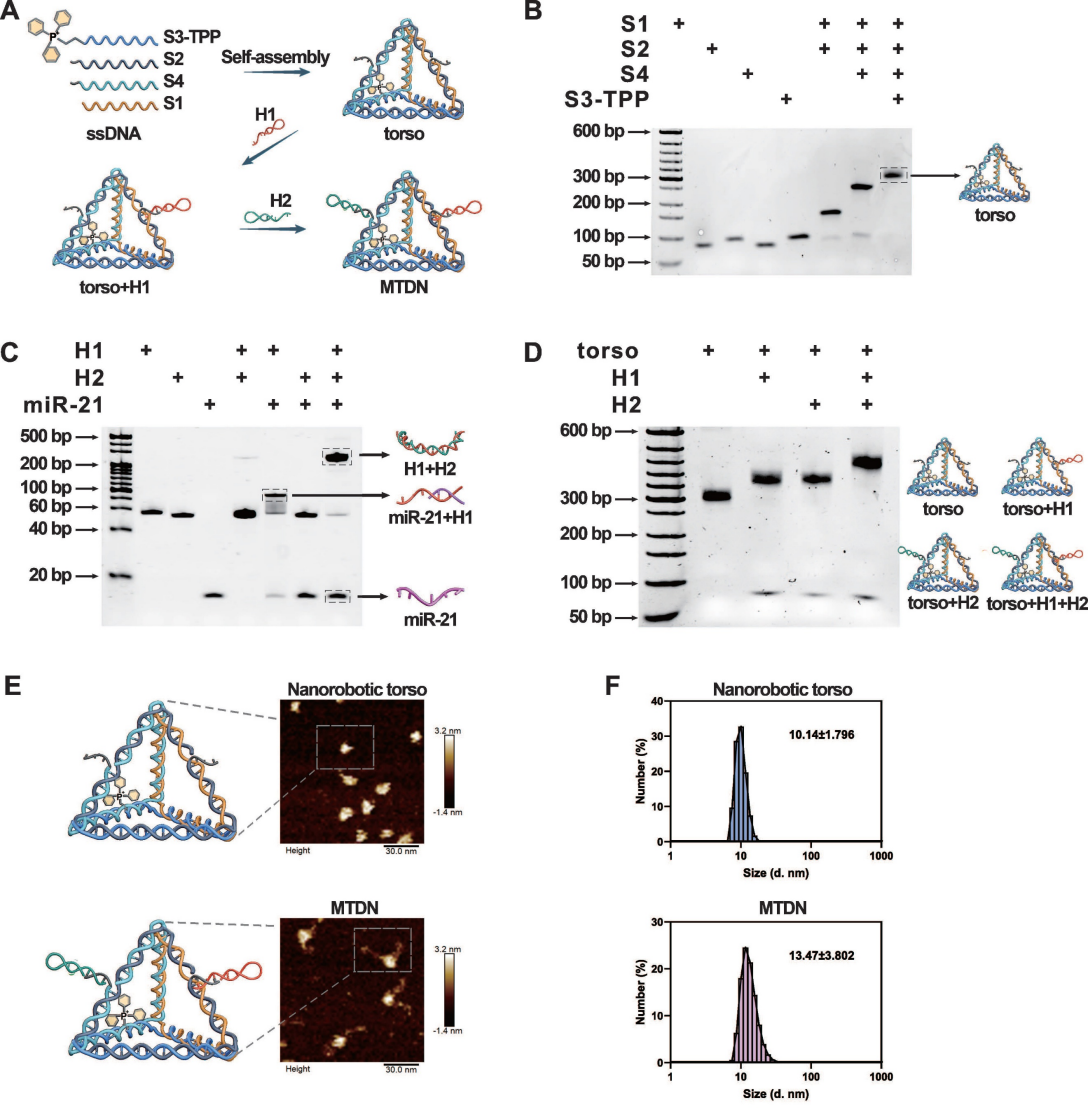
Self-assembly and characterization of MTDN. (A) Schematic illustration of the stepwise assembly process of MTDN. (B) Native PAGE analysis of the self-assembly process of MTDN's torso. (C) Feasibility analysis of the CHA detection system using native PAGE. H1 and H2: 200 nM, target miR-21: 100 nM. (D) Verification of the linking process between MTDN and two hairpins (H1 and H2) via native PAGE characterization. (E) AFM images of MTDN's torso and the MTDN. Scale bar: 30 nm. (F) Diameter size distribution of MTDN's torso and MTDN measured by dynamic light scattering (DLS).

The core component of MTDN, the torso structure, was engineered through the assembly of four complementary single-stranded DNAs (S1-S4) based on Watson-Crick base pairing principles. The 5′ end of ssDNA S3 was functionalized with TPP to endow mitochondria-targeting capability to nanorobots. The complete assembly strategy of MTDN was illustrated in Figure 1A, in which the designed oligonucleotide sequences were detailed in Table S1. The assembly process was monitored by native PAGE analysis, which revealed a characteristic stepwise assembly process as shown in lines 5-8. This progressive decrease in electrophoretic mobility indicated increasing molecular complexity, providing direct evidence for the successful formation of the polyhedral torso structure (Figure 1B). In addition, native PAGE analysis demonstrated the detection capability of the MTDN′ arm. Lanes 5-8 on gel revealed a sequential assembly process: initial binding of the target with H1, followed by H1-H2 hybridization, producing a high-molecular-weight complex (Figure 1C). Subsequently, two functional arms (H1 and H2) were sequentially conjugated to MTDN's torso, completing MTDN assembly. In an exhibition of lane 5 on the native PAGE gel, the fully assembled MTDN demonstrated significantly reduced electrophoretic mobility because the graft of arms enabled the three-dimensional structure to get more sophisticated (Figure 1D). The structural characterizations of MTDN were investigated using multiple analytical techniques. Atomic force microscopy (AFM) revealed the six edges of the DNA tetrahedron framework and its corresponding three-dimensional geometry (Figure 1E, top panel), as well as the morphological changes following arm conjugation (Figure 1E, bottom panel). Dynamic light scattering (DLS) analysis demonstrated a monodisperse size distribution, confirming the homogeneity of MTDN. Notably, the mean hydrodynamic diameter increased from 10.14 nm to 13.47 nm upon arm attachment (Figure 1F). These comprehensive characterization results collectively validate the precise assembly of MTDN architecture. Upon responding to miR-21, the MTDN was characterized using AFM (Figure S1) and native PAGE analysis (Figure S2). The results revealed partial aggregation of MTDN and a reduced mobility shift after MTDN hybridized with the target, compared to the target-free reaction. These findings indicate the target detection capability of MTDN.

### Optimal condition and performance

**Figure 2:**
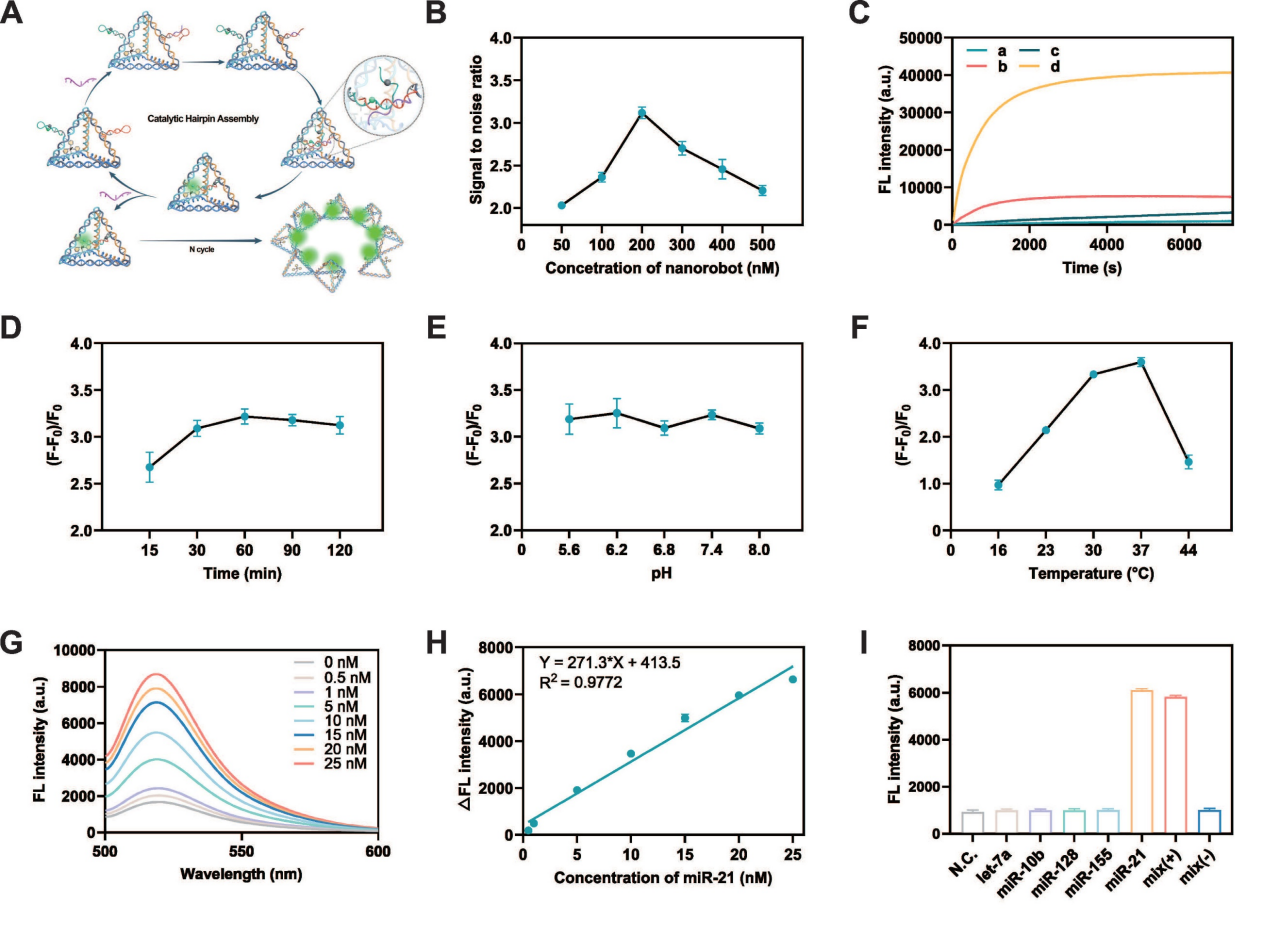
Evaluation of detection conditions and performance of MTDN. (A) Schematic illustration of the detection principle of MTDN. (B) Signal-to-noise ratio (SNR) of the MTDN system at gradient concentration of MTDN (ranging from 50 nM to 500 nM). (C) Real-time monitoring of the detection system in response to the target. Free CHA detection system (curves a/b) and MTDN detection system (curves c/d) reacted with the target (curves a and c: 0 nM miR-21; curves b and d: 20 nM miR-21). (D) Determination of the optimal response time in the endpoint assay by comparing the SNR at different time points. (E) Investigation of the influence of pH on detection performance by comparing the SNR at varying pH values. (F) Exploration of the optimal response temperature by comparing the SNR at different temperatures. (G) Fluorescence spectra of MTDN (200 nM) after reacting with different concentrations of miR-21 (ranging from 0 nM to 25 nM). (H) Linear relationship between fluorescence signals of MTDN and miR-21 concentrations. The linear formula: Y = 271.3X + 413.5 (R² = 0.9772). (I) Selectivity of MTDN toward different types of miRNAs (miR-21, let-7a, miR-10b, miR-128, miR-155), positive mixture, and negative mixture. N.C. (no miRNAs), Mix (-) denotes a miRNA mixture without miR-21 (let-7a: miR-10b: miR-128: miR-155 = 1:1:1:1), and Mix (+) denotes a miRNA mixture with miR-21 (let-7a: miR-10b: miR-128: miR-155: miR-21 = 1:1:1:1:1). Data are presented as mean ± SD, n=3.

The MTDN, labeled with FAM and BHQ1 on H2, was prepared to enable precise quantification of the target miRNA concentration. During the assembly of MTDN, the close spatial proximity of FAM and BHQ-1 facilitated efficient fluorescence quenching. Upon specific recognition and binding of the target miRNA by H1, a conformational rearrangement in the MTDN structure was triggered, leading to the separation of FAM and BHQ-1. This structural transition effectively eliminated the quenching effect of FAM fluorescence. The target miRNA remains unconsumed during the MTDN detection process enabling it to initiate multiple catalytic cycles. This unique feature allows for the continuous generation of fluorescent MTDN, leading to significant signal amplification (Figure 2A).

In the optimization experiments for the MTDN system, the signal-to-noise ratio (SNR) was calculated by comparing the fluorescence intensity in the presence of 20 nM miR-21 (signal) to that in the absence of miR-21 (noise). Initially, the optimal concentration of MTDN was determined by comparing the SNR at various concentrations. The results indicated that the SNR reached its maximum at an MTDN concentration of 200 nM (Figure 2B). Real-time fluorescence monitoring of both the free CHA system and the MTDN system revealed that, at the same probe concentration (200 nM), the MTDN system exhibited superior signal intensity and a faster, more sensitive response to miR-21 (Figure 2C). This enhancement is attributed to the spatial confinement effects within the MTDN structure. Kinetics analysis revealed that the fluorescence intensity rapidly increased within the first 30 min and reached a plateau after 1 h (Figure 2C), which aligned with the observation of the highest SNR at different response times (Figure 2D). This indicates that the optimal reaction time for endpoint detection is 1 h. Further investigations into the impact of pH variations showed that changes in pH did not significantly affect the hybridization efficiency or the detection performance of the MTDN system (Figure 2E). Furthermore, the highest signal-to-noise ratio (SNR) was observed at 37°C, demonstrating the compatibility of MTDN with physiological environments and their robust performance for in situ imaging applications (Figure 2F).

The sensitivity of the MTDN system was rigorously assessed under the aforementioned optimized conditions (the reaction temperature: 37°C, time: 1h, and pH value: 7.4). A clear increase in fluorescence intensity was observed as the concentration of miR-21 increased, showing a linear relationship from 0.5 nM to 25 nM. The linear equation is Y = 271.3X +413.5 (R^2^ = 0.9772) (Figure 2H). The limit of detection (LOD) of MTDN was 0.26 nM according to the 3σ/slope rule. To evaluate specificity, the MTDN system was tested against miR-21, four homologous miRNAs (let-7a, miR-10b, miR-128, and miR-155), a positive mixture containing all these miRNAs including miR-21, and a negative mixture excluding miR-21. The results revealed a significant fluorescence increase exclusively in the presence of miR-21, while other conditions yielded signals comparable to background levels. This confirms the exceptional specificity of the MTDN system and its potential for accurate and selective in situ detection of miR-21 (Figure 2I). These findings highlight the precision of the MTDN system for targeted miRNA detection.

### MTDN system for spatial mitomiR-21 imaging in live cells and breast cancer diagnosis

**Figure 3:**
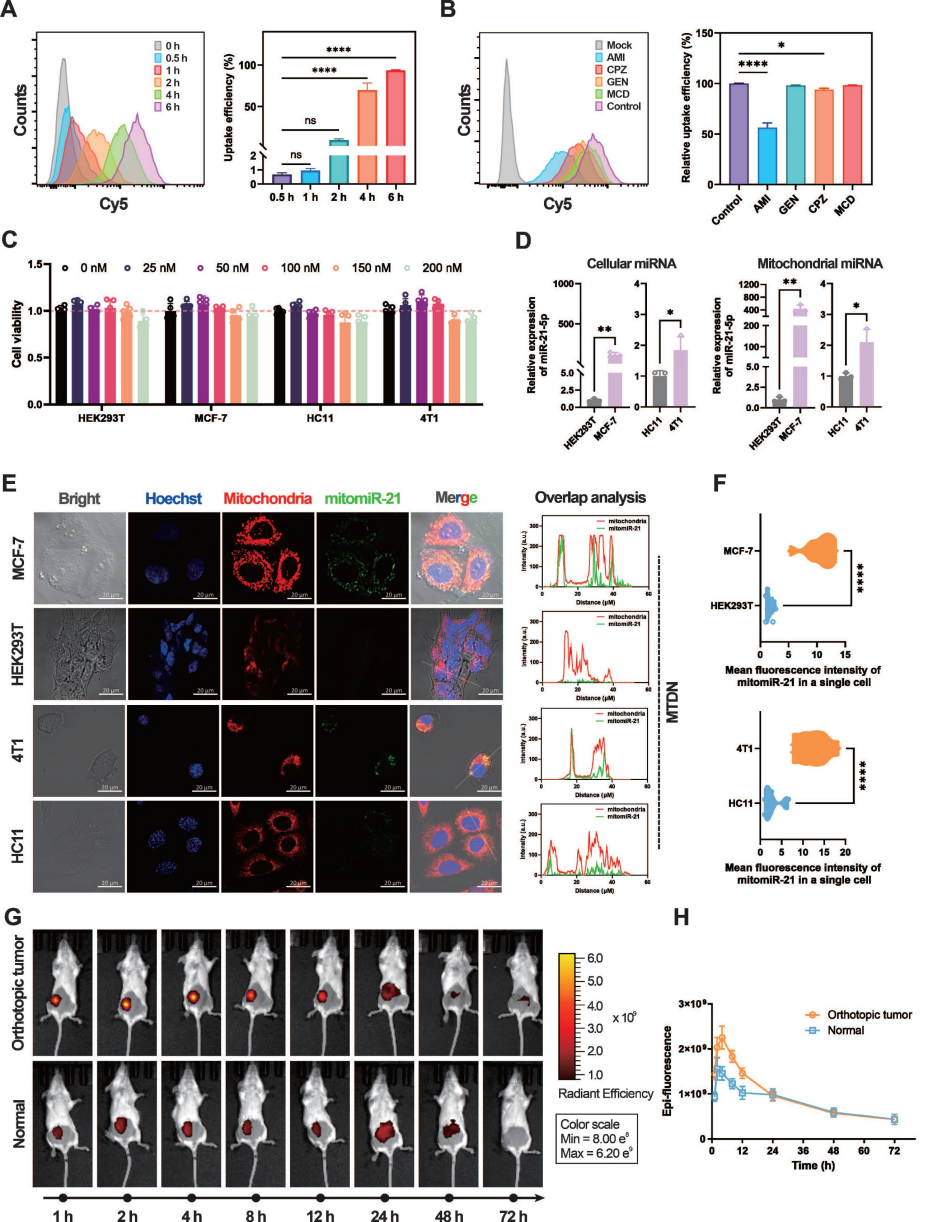
Analysis of the endocytosis mechanism and mitochondrial imaging capability of MTDN. (A) Representative flow cytometry images of MTDN uptake by MCF-7 cells at different incubation times (0, 0.5, 1, 2, 4, and 6 h) (left panel), and statistical analysis (n = 3) of the relative fluorescence intensity of cells (right panel). Data are presented as mean ± SD (n = 3). (****p < 0.0001; ns, not significant). (B) Flow cytometry images of MCF-7 cells incubated with MTDN for 6 h after treatment with different endocytosis inhibitors (left panel). Statistical analysis is shown on the right (n = 3). Data are presented as mean ± SD (n = 3). (*p < 0.05; ****p < 0.0001; ns, not significant). (C) Cytotoxicity assay of four cell types (HEK293T, MCF-7, HC11, and 4T1 cells) incubated with MTDN at various concentrations (0 to 200 nM) for 24 h. Data are presented as mean ± SD (n = 5). (D) Relative expression of cellular miR-21 and mitochondrial miR-21 in four cell lines measured by RT-qPCR. Data are presented as mean ± SD (n = 3). (E) Confocal images of HEK293T, MCF-7, HC11, and 4T1 cells using MTDN to detect mitochondrial miR-21. Scale bars: 20 μm. Overlap analysis indicates the degree of fluorescence colocalization of mitochondria (red) and miR-21 (green) along the white line using ImageJ software. (F) Mean fluorescence intensity of mitomiR-21 in single cells from breast cancer and normal cells, analyzed using ImageJ software (n = 9). Data are presented as violin plots, showing the median and quartiles (n = 15) (****p < 0.0001). (G) Representative three-dimensional live epi-fluorescence images acquired at 1, 2, 4, 8, 12, 24, 48, and 72 h after injecting diagnostic MTDN into healthy mice and mice with orthotopic breast cancer. (H) Time-dependent epi-fluorescence intensity kinetics curve measured using Living Image software. Data are presented as mean ± SD (n = 3).

The biocompatibility and cellular uptake mechanisms of MTDN were evaluated to ensure their stability and functionality in mitochondria-targeted live cell imaging and diagnosis *in vivo*. In the assessment of MTDN stability, native PAGE gel electrophoresis revealed minimal degradation after an 8-h incubation period in 10% FBS, remaining detectable even after a 12-h reaction period. Conversely, free hairpins exhibited substantial degradation within 2 h. (Figure S3), showing that the tetrahedral framework structure of MTDN significantly minimizes signal leakage resulting from the biodegradation of functional hairpins. Furthermore, the endocytosis kinetics were investigated by monitoring the uptake of Cy5-labeled MTDN at various time intervals (0, 0.5, 1, 2, 4, and 6 h). The results indicated that 93.4% of cells internalized MTDN within a 6-h incubation period (Figure 3A). Additional experiments employing specific endocytosis inhibitors to interfere with endocytosis revealed that AMI (an inhibitor of macropinocytosis) significantly reduced MTDN uptake, while CPZ (a clathrin-mediated endocytosis inhibitor) had a moderate effect (Figure 3B). These findings suggest that MTDN is primarily internalized via macropinocytosis and caveolin-mediated endocytosis. The biocompatibility of MTDN was evaluated after treating cells with varying concentrations of MTDN for 24 h. The results showed no significant reduction in cell viability (Figure 3C), confirming that the MTDN exhibits high biocompatibility and is well-suited for both *in vitro* and *in vivo* detection applications.

Quantitative real-time PCR (RT-qPCR), recognized as the gold standard, was employed to measure mitochondrial miR-21 (mitomiR-21) expression levels in both normal and tumor cells before utilizing MTDN for in situ imaging of mitomiR-21. To extract mitochondrial RNAs, mitochondria were first isolated from live cells. Images of biological transmission electron microscopy (Bio-TEM) verified the isolated mitochondria have intact mitochondrial structures and high purity (Figure S4). Subsequent RT-qPCR showed that both cellular and mitochondrial miR-21 levels were higher in cancer cells (MCF-7/4T1 cells) than in normal cells (HEK293T/HC11 cells) (Figure 3D). The mitochondria-targeting capability was evaluated by analyzing confocal microscopy images of mitochondria-untargeted DNA nanorobots (UTDN) and MTDN in in-situ imaging experiments. Imaging with UTDN in cancer cells (MCF-7/4T1) revealed detective signals (green) localized in the cytoplasm. Overlap analysis showed that these signals (green) exhibited no significant colocalization with mitochondria (red, stained with Mito-Tracker CMXRos, a mitochondria-specific dye), confirming the detection of cellular miR-21(Figure S5). Conversely, imaging with MTDN demonstrated a high degree of colocalization between the detective signals (green) and mitochondria (red), validating the successful detection of mitomiR-21 and the mitochondria-targeting capability (Figure 3E). Statistical analysis further revealed significantly higher mean fluorescence intensity in the mitochondria of cancer cells (MCF-7/4T1) than in normal cells (HEK293T/HC11) when using MTDN (Figure 3F), consistent with the results obtained from RT-qPCR. These findings confirm that MTDN for detecting mitomiRNA exhibits excellent evaluation performance.

Motivated by the live imaging results, we investigated the potential of MTDN for *in vivo* breast cancer diagnosis. When orthotopic breast tumors reached a volume of 100 mm³, MTDN (500 nM, 100 μL) were intratumorally injected into the mice. For comparison, the same dose was administered to the mammary glands of healthy mice as a control group. Epi-fluorescence was monitored continuously for 72 h post-injection (Figure 3G) to analyze the kinetics. The results demonstrated a rapid increase in epi-fluorescence intensity in the cancer group, peaking at 4 h post-injection, with an intensity 1.55-fold higher than that of the normal group. By 24 h, MTDN no longer accumulated in the tumor area, showing diffusion and metabolism, with no significant difference in epi-fluorescence intensity between the two groups thereafter. These findings demonstrate that the MTDN is effective for *in vivo* cancer diagnosis (Figure 3H).

### Intracellular mitochondrial accumulation of Dox after using MTDN delivery

**Figure 4:**
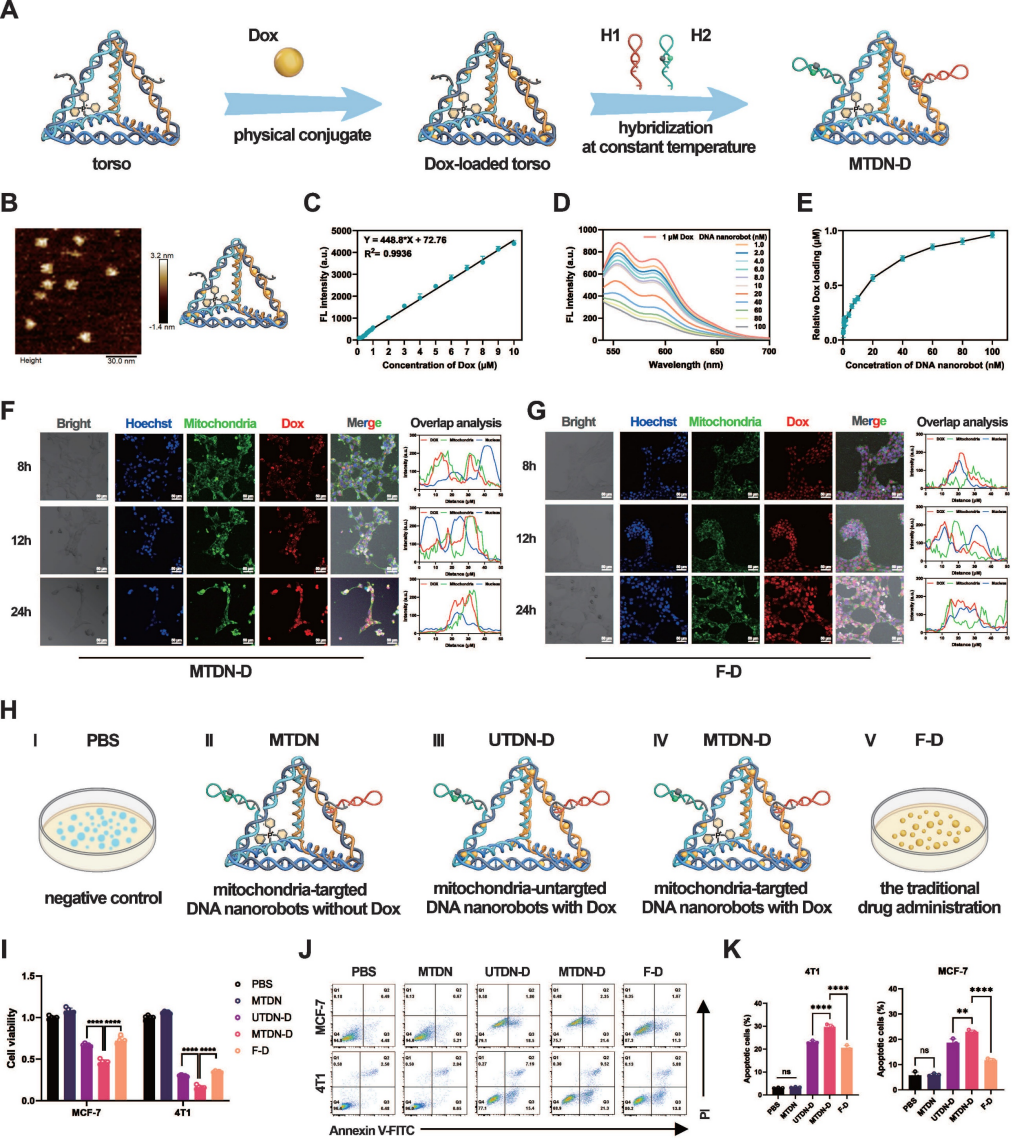
Loading, intracellular release, and *in vitro* antitumor effects of Dox. (A) Schematic illustration of the procedure of Dox loading into MTDN (Figure 4A). (B) AFM image of the MTDN's torso loaded with Dox. Scale bar: 30 nm. (C) Linear relationship between the concentration of free Dox (X) and fluorescence intensity (Y). (D) Fluorescence spectra of residual Dox after titrating 1 µM Dox with varying concentrations of MTDN. (E) Dynamic curve illustrating the relationship between loaded Dox concentration and MTDN concentration. (F) Intracellular release and distribution of Dox delivered by MTDN (MTDN-D) at 8, 12, and 24 h. Scale bars: 50 nm. Overlap analysis indicates the degree of fluorescence colocalization of Dox (red), mitochondria (green), and the nucleus (blue) along the white line using ImageJ software. (G) Intracellular distribution of free Dox (F-D) at 8, 12, and 24 h. Scale bars: 50 nm. Overlap analysis indicates the degree of fluorescence colocalization of Dox (red), mitochondria (green), and the nucleus (blue) along the white line using ImageJ software. (H) Schematic of different drug groups. PBS: a negative control; MTDN: mitochondria-targeted DNA nanorobots without Dox; UTDN-D: mitochondria-untargeted DNA nanorobots with Dox; MTDN-D: mitochondria-targeted Dox with Dox; F-D: free Dox, the traditional drug administration. (I) Cell proliferation viability of MCF-7 and 4T1 cells treated with PBS, MTDN, UTND-D, MTDN-D, or F-D for 24 h, assessed using the CCK-8 kit. The equivalent concentration of Dox was 2 µM for MCF-7 cells and 4 µM for 4T1 cells. (J) Representative flow cytometry images of cell apoptosis analysis after 24 h of treatment with PBS, MTDN, UTND-D, MTDN-D, or F-D. (K) Statistical analysis of the apoptotic cell rates in each group (n = 3). Data are presented as mean ± SD (n = 3). (**p < 0.01; ****p < 0.0001; ns, not significant).

Given its exceptional mitochondria-targeting capability, we employed MTDN as an organelle-specific drug carrier for synchronous mitochondrial regulation. The schematic outlines the procedure for loading dox into MTDN (Figure 4A). AFM images confirmed that the tetrahedral framework of MTDN retained its spatial structure after loading Dox (Figure 4B). The fluorescence spectrum analysis and titration experiments were conducted to evaluate the Dox-loading capability of MTDN. Initially, we explored the correlation between fluorescence intensity (Y) and Dox concentration (X) by analyzing the fluorescence spectra of free Dox at various concentrations. The linear regression equation is Y = 448.8X + 72.76 (R² = 0.9936) (Figure 4C and S6). In the titration experiment, Dox at a final concentration of 1 µM was incubated with varying concentrations of MTDN at 37 °C for 12 h. Subsequently, the fluorescence spectra of the residual Dox were recorded (Figure 4D), and its concentration (C _(residual Dox)_) was calculated using the aforementioned linear regression formula (Figure 4C). The dynamic relationship between the concentration of loaded Dox (C_ (Dox)_ = 1 µM - C _(residual Dox)_) and that of MTDN was determined, revealing that the dynamic curve plateaued at a concentration ratio of 16.67:1 (C_ (Dox)_: C_(MTDN)_) (Figure 4E). Following Dox loading onto the torso of UTDN or MTDN, arms were grafted to form the Dox-loaded UTDN (UTDN-D) or the Dox-loaded MTDN (MTDN-D). The successful loading of Dox was further validated by native PAGE analysis, where bands corresponding to MTDN-D and UTDN-D appeared at higher molecular weights, as shown in lanes 4 and 7 (Figure S7). These results demonstrate that MTDN serves as an effective Dox carrier.

We further investigated the intracellular release and distribution of Dox delivered by MTDN. Following MTDN-D treatment, confocal imaging analysis revealed that Dox fluorescence (red) exhibited significant colocalization with mitochondria (green, stained with MitoTracker Green, a mitochondria-specific dye). After 24 h of incubation, partial colocalization of Dox fluorescence (red) with the nucleus (blue) was observed. These results indicate that when Dox is delivered via MTDN, it largely accumulates in mitochondria and is subsequently transported to the nucleus over time (Figure 4F and S8). In contrast, in cells treated with Free Dox (F-D), Dox fluorescence (red) showed strong overlap with the nucleus (blue) after 8 and 12 h of incubation. By 24 h, partial overlap of Dox fluorescence (red) with mitochondria (green) was observed. This suggests that, in the F-D treatment group, Dox is primarily enriched in the nucleus before being released into the cytoplasm (Figure 4G).

The effects of MTDN-D-induced mitochondrial Dox accumulation were evaluated using CCK-8 and apoptosis assays. For effective control of variables, the experiments were divided into five groups: PBS (negative control), MTDN, UTDN-D, MTDN-D, and F-D (free Dox, representing traditional drug administration) (Figure 4H). The UTDN-D, MTDN-D, and F-D groups contained equivalent concentrations of Dox, while the MTDN, UTDN-D, and MTDN-D groups contained equivalent concentrations of DNA nanorobots. The CCK-8 assay results demonstrated a significant enhancement in proliferation inhibition when UTDN or MTDN were utilized for Dox delivery (UTDN-D or MTDN-D groups vs. F-D group), which can be attributed to the increased intracellular retention of Dox. Notably, the MTDN-D treatment group exhibited the lowest cell viability, suggesting that mitochondrial accumulation of Dox significantly augmented the therapeutic efficacy (Figure 4I). Flow cytometry-based apoptosis analysis further revealed a marked increase in apoptotic cells after 24 h of MTDN-D treatment compared to other groups (Figure 4J and 4K). Notably, no significant increase in apoptosis or reduction in cell viability was observed in the MTDN group. These findings demonstrate that MTDN is safe and effective in mitochondria-targeting Dox delivery, and it significantly enhances anti-tumor efficacy in vitro caused by mitochondrial accumulation of Dox.

### Intrinsic apoptosis pathway activated by MTDN-D-mediated mitochondria damage

**Figure 5:**
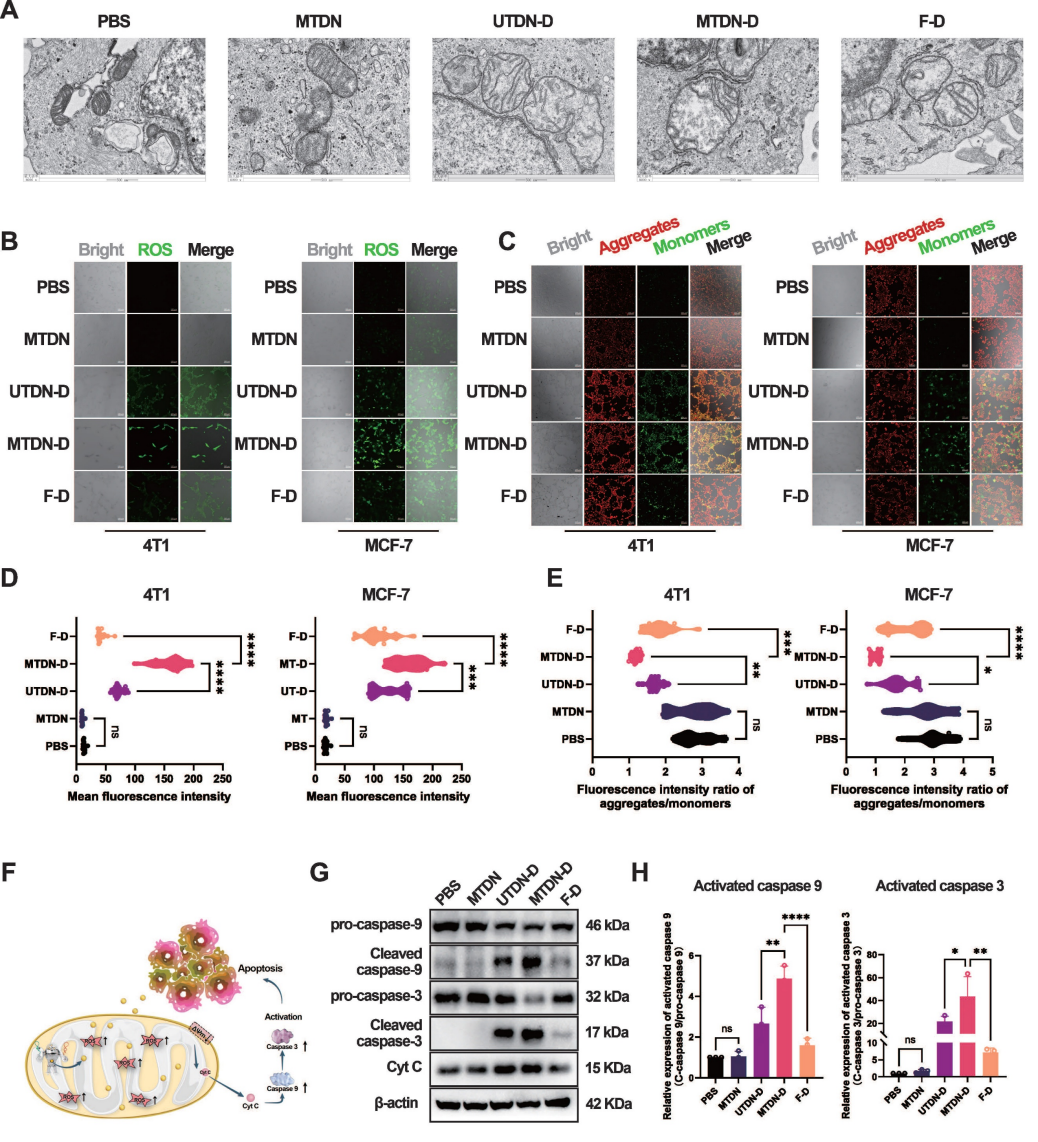
Activation of mitochondria-mediated intrinsic apoptosis pathways. (A) Biological transmission electron microscopy (Bio-TEM) images of MCF-7 cells treated with five different conditions. Scale bars: 500 nm. (B) Confocal images of ROS levels using DCFH-DA probes after treatment for 24 h. Scale bars: 100 μm. (C) Fluorescence images of mitochondrial aggregates (red) and monomers (green) stained with the JC-1 probe. Scale bars: 100 μm. (D) Quantitative analysis of ROS fluorescence intensity. Data are presented as violin plots, showing the median and quartiles (n = 15) (***p < 0.001; ****p < 0.0001; ns, not significant). (E) Evaluation of mitochondrial membrane potential (MMP) based on the fluorescence intensity ratio (red/green). Data are presented as violin plots, showing the median and quartiles (n = 15) (*p < 0.05; **p < 0.01; ***p < 0.001; ****p < 0.0001; ns, not significant). (F) Schematic of the intrinsic apoptotic pathway activated by MTDN-D treatment. (G) Western blot (WB) images showing the expression levels of proteins in the Cytc/caspase-9/caspase-3 signaling pathway in 4T1 cells treated with PBS, MTDN, UTND-D, MTDN-D, or F-D for 24 h. (H) Statistical analysis of the expression levels of activated caspase-9 and caspase-3 (activated caspase-9 = cleaved caspase-9/pro-caspase-9; activated caspase-3 = cleaved caspase-3/pro-caspase-3). Data are presented as mean ± SD (n = 3). (*p < 0.05; **p < 0.01; ***p < 0.001; ****p < 0.0001 ns, not significant).

Mechanistically, bio-TEM technology was employed to characterize mitochondrial morphology in MCF-7 cells after treatment with PBS, MTDN, UTDN-D, MTDN-D, and F-D for 24 h. The images revealed significant mitochondrial alterations specifically in the MTDN-D treatment group, including a marked reduction in the long-to-short diameter ratio, loss of internal structural integrity, and pronounced swelling, compared to other groups (Figure 5A). These morphological changes are likely attributable to Dox accumulation in mitochondria, which may induce excessive ROS generation and a sharp decline in mitochondrial membrane potential (MMP). To further investigate, intracellular ROS levels were measured using a ROS probe (DCFH-DA). Confocal imaging (Figure 5B) and fluorescence intensity analysis (Figure 5D) demonstrated significantly elevated ROS levels in the MTDN-D treatment group compared to others. Additionally, mitochondrial aggregates (red) and monomers (green) were labeled using the JC-1 probe and visualized via confocal imaging after treatment for 24 h (Figure 5C). The intensity ratio of red to green fluorescence indicated a sharp decline in mitochondrial membrane potential (MMP) in the MTDN-D treatment group (Figure 5E). In addition, mitochondrial damage induced by MTDN-D treatment triggered the release of cytochrome C (Cyt C), activating the intrinsic apoptosis pathway (Figure 5F). Western blot analysis demonstrated a significant upregulation in the relative expression of activated caspase-9 and caspase-3 compared to other groups, with the relative expression of activated proteins calculated as the ratio of cleaved proteins to pro-proteins (Figure 5G and H). These findings underscore the potential of MTDN-D in antitumor applications through the activation of the intrinsic apoptosis pathway.

### Antitumor therapy through enhanced mitochondrial damage mediated by MTDN-D treatment *in vivo*

**Figure 6:**
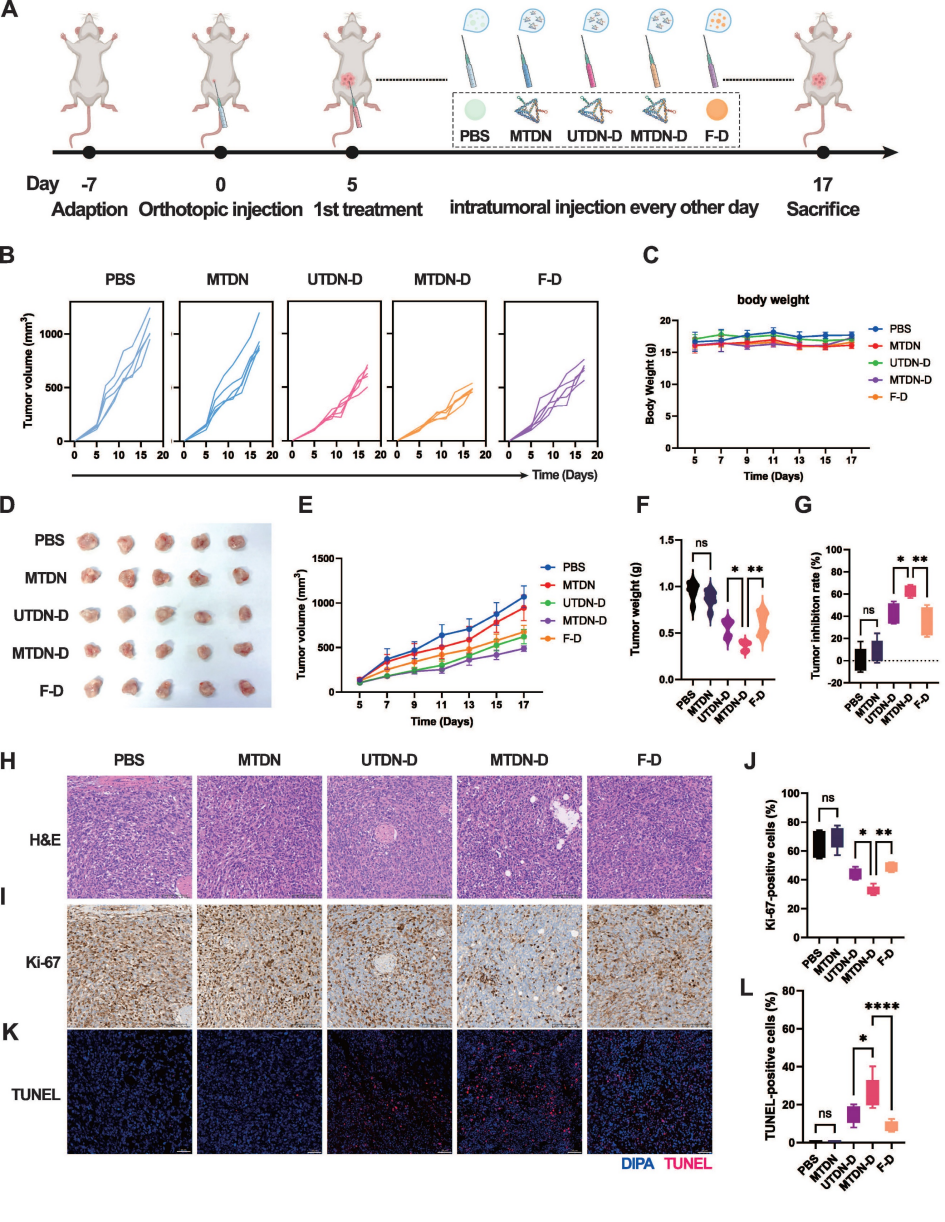
MTDN-D treatment to enhance antitumor efficacy *in vivo*. (A) Schematic of the antitumor therapy design using orthotopic mouse models of mammary tumors. Tumor-bearing mice received intratumoral injections of PBS, N, UTN-D, N-D, or F-D every other day for two weeks. (B) Analysis of individual tumor growth kinetics of each mouse in five treatment groups (five mice per group). (C) Body weight monitoring curves for mice in different treatment groups. (D) Photographs of tumors excised from the mouse mammary gland after two weeks of treatment. (E) Overall tumor growth curves following treatment with different drug formulations. Data are presented as mean ± SD (n = 5). (F) Tumor weight after two weeks of diverse treatments. Data are presented as mean ± SD (n = 5). (*p < 0.05; **p < 0.01; ns, not significant). (G) Relative tumor inhibition rates for the five treatment groups, with the PBS group as the control. (*p < 0.05; **p < 0.01; ns, not significant). (H) H&E staining analysis of paraffin-embedded tissue sections from orthotopic tumors. (I) Representative immunohistochemical images of Ki-67 in tumor tissue sections. (J) Statistical analysis of the Ki-67-positive rate using ImageJ software (n = 5). (*p < 0.05; **p < 0.01; ns, not significant). (K) Representative immunohistochemical fluorescence images of TUNEL in tumor tissue sections, where blue fluorescence indicates cell nuclei and red fluorescence denotes TUNEL-positive cells. (L) Fluorescence intensity and TUNEL-positive rate analysis using ImageJ software (n = 5).

Given the remarkable antitumor efficacy of MTDN-D *in vitro*, we evaluated its therapeutic potential in female BALB/c mice with orthotopic breast cancer. The mice were administered intratumoral injections of PBS, MTDN, UTDN-D, MTDN-D, or F-D as the tumor volume reached approximately 100 mm³, respectively. Throughout the process, body weight and tumor volume were recorded every other day. After two weeks of treatment, the mice were sacrificed and analyzed (Figure 6A). Continuous monitoring of the tumor volume of each mouse revealed that tumor growth was significantly suppressed in the groups treated with equal concentrations of Dox (UTDN-D, MTDN-D, and F-D group) compared to the PBS group. Although tumor growth kinetics showed a slight decline in the MTDN group, no significant differences were observed compared to the PBS group (Figure 6B). Body weight monitoring curves indicated that the growth of the mice was not adversely affected by the administered drugs (Figure 6C). Notably, the MTDN group had no significantly negative impact on mouse growth compared to the PBS group, highlighting the favorable biosafety profile of MTDN as a drug delivery platform. At the end of the antitumor therapy, tumors were excised from the mice (Figure 6D), and their weights were measured. Consistent with the overall tumor growth (Figure 6E), the lightest tumor weight (Figure 6F) was observed in the MTDN-D group. The tumor inhibition efficiencies of MTDN, UTDN-D, MTDN-D, and F-D were 8.5%, 41.6%, 64%, and 35%, respectively, compared to the PBS group (Figure 6G).

Following paraffin fixation and embedding, tissue sections were subjected to H&E staining (Figure 6H) to identify cancer cells and delineate tumor regions. Tumor cell proliferation activity was assessed using Ki-67 immunohistochemistry (Figure 6I), and apoptosis was evaluated via TdT-mediated dUTP nick-end labeling (TUNEL) immunofluorescence (Figure 6J). The proportion of Ki-67-positive cells significantly decreased in the groups treated with equal concentrations of Dox (UTDN-D, MTDN-D, and F-D), with no notable changes observed between the PBS and MTDN groups (Figure 6K). The lowest Ki-67-positive rate was observed in the MTDN-D group, indicating enhanced inhibition of tumor proliferation by MTDN-D treatment. Meanwhile, the ratio of TUNEL-positive cells markedly increased in the groups treated with equal concentrations of Dox (UTDN-D, MTDN-D, and F-D), with no significant changes observed between the PBS and MTDN groups (Figure 6L). The highest TUNEL-positive rate was observed in the MTDN-D group, demonstrating that Dox-induced apoptosis was significantly enhanced through MTDN delivery.

The safety of this therapy was evaluated using blood samples and major organs (heart, liver, spleen, lung, and kidney) from the treated mice. The hematological indices derived from the serum biochemical parameters (Figure S9) in mice subjected to the different antitumor therapy consistently remained within the normal physiological range. Histological examination revealed no significant structural damage to the major organs, except for cardiac tissue in the F-D group. In this group, slight atrophy of myocardial cells was observed, attributed to Dox-induced increased oxidative stress and apoptosis in cardiomyocytes following its accumulation in cardiac tissue (Figure S10). However, owing to the high stability and cellular uptake efficiency of the DNA tetrahedral framework in UTDN/MTDN, these nanorobots effectively enhance the retention of DOX in tumor cells while mitigating damage to normal tissues caused by the rapid increase in blood concentration of DOX during drug administration. These findings robustly demonstrate the safety and therapeutic efficacy of MTDN as an innovative mitochondria-targeted drug delivery system for intratumoral administration of Dox in breast cancer treatment.

## Discussion

In this study, we developed a multipurpose MTDN-based system for mitomiRNA-targeted imaging and mitochondria-targeted drug delivery, enabling highly sensitive detection of tumor mitomiR-21 and synergistic enhancement of anti-tumor efficacy. Utilizing their unique mitochondria-targeting capability, MTDN efficiently delivered imaging probes to the mitochondria of tumor cells, achieving highly sensitive detection of mitochondrial markers. Upon loading Dox, MTDN efficiently targets and releases it to mitochondria in cancer cells, inducing ROS-mediated mitochondria damage and activating the intrinsic apoptotic pathway.

Specific mitochondria-targeted imaging sensors and drug carriers can be achieved by conjugating highly stable TPP with nanomaterials 24. The elevated mitochondrial membrane potential (ΔΨm) 25 in cancer cells facilitates the targeting and penetration of TPP-conjugated nanomaterials across the mitochondrial membrane, as the mitochondria-targeting capability of TPP is primarily driven by its hydrophobicity and the polarity of its phosphonium group. Extensive research has demonstrated that the nanomaterials' intracellular stability and endocytosis pathways are influenced by their size, shape, and charge 26, 27.

Thus, our MTDN is rapidly internalized by cancer cells and exhibits a significantly higher mitochondrial co-localization rate through choosing a suitable tetrahedral size as the TPP-functionalized framework. Previous studies have shown that nanosensors based on tFNA enhance local probe concentration and accelerate reaction speed and efficiency through the spatial confinement effect of their frame structure features 28, 29. In our design, the nick and extension of the edges of the tetrahedral framework to connect functional probes, further enhance the spatial confinement effect and improve the reaction kinetics of MTDN. Owing to the excellent programmability of MTDN, multiple imaging probes can be incorporated by customizing the functional hairpin sequences, enabling targeted detection of different mitochondrial miRNAs. Compared to traditional imaging methods 30, 31, MTDN represents an advanced detection platform that elevates detection precision from the cellular to the subcellular level. Those characteristics have the potential for real-time monitoring of mitochondrial functional status and metabolic abnormalities in tumor cells, providing accurate diagnostic information to guide treatment strategies.

Therapeutically, precise delivery of Dox to the mitochondria of tumor cells can significantly enhance its anti-tumor efficacy 32, 33, while reducing systemic toxicity 34. This may be attributed to the accumulation of Dox in mitochondria, which induces excessive ROS production, amplifying its cytotoxic effects and potentially activating tumor immune responses 35. Our study revealed that Dox accumulation in mitochondria was markedly increased in MTDN-D-treated cells. That leads to mitochondrial damage characterized by swelling, ROS overproduction, and decreased mitochondrial membrane potential, ultimately activating the intrinsic apoptotic pathway. To address the dose-dependent cardiotoxicity of Dox during anti-tumor treatment 36, numerous nanocarriers have been developed to deliver Dox, effectively mitigating Dox-induced cardiotoxicity 37. In our study, Dox carried by MTDN to intratumoral treatment effectively increases the release of Dox in tumors and avoids the sharp rise in blood concentration. That not only improved the tumor inhibition rate but also significantly reduced toxicity to normal tissues, particularly the heart, during antitumor therapy.

## Conclusions

Collectively, MTDN enables simultaneous detection of mitomiRNA and mitochondria-targeted delivery of Dox, achieving integrated diagnosis and treatment. With its sensitive precision detection capabilities and enhanced therapeutic efficacy, this multifunctional platform offers innovative strategies and methodologies for precise tumor therapy, holding significant potential for real-time monitoring of treatment effects.

## Materials and Methods

### Materials

TE buffer (pH 7.4) was used to dissolve oligonucleotides (Table S1) which were synthesized and purified by high-performance liquid chromatography. The TAMg buffer for MTDN construction was prepared using Tris (40 mM) and MgCl₂·6H₂O (7.6 mM), with acetic acid added to adjust the pH to 7.4. The above reagents were acquired from Sangon Biotech Co., Ltd. (Shanghai, China). DNA markers (20 bp ladder, 50 bp ladder) were procured from Takara Biomedical Technology Co., Ltd. (Beijing, China). Cells (MCF-7, HEK293T, HC11, and 4T1) were sourced from the American Type Culture Collection (ATCC). Hoechst 33342 staining solution, cell counting kit-8 (CCK-8), MitoTracker Green, and MitoTracker Red CMXRos were obtained from Beyotime Co., Ltd. (Shanghai, China).

### Preparation of the MTDN

The MTDN consists of six customized DNA strands (S1-S4 and two hairpins), with sequences detailed in Table S1. Initially, four DNA strands (S1, S2, S4, and S3-TPP) were mixed in an equal molar ratio (1.2 μmol each) in TAMg buffer. The tetrahedral torso with the command center was constructed by heating at 95 °C for 5 min, maintaining a cooldown at 54 °C for 30 min, and then rapidly cooling to 4 °C. Concurrently, the two hairpins were denatured by heating at 95 °C for 10 min, maintained a cooldown at 25 °C for 1 h, and further chilled to 4 °C. Subsequently, an equal amount of the two hairpins (H1 and H2) and the tetrahedral torso were mixed and incubated at 37 °C for 30 min to assemble the MTDN. The morphology of the products was characterized using atomic force microscopic (AFM). The mica substrate was prepared by depositing 80 μL of 1% (V/V) APTES for 5 min, followed by the use of 20 μL TAMg buffer to remove residual fluids. The mica substrate was then dried under nitrogen protection before adding 20 μL of the sample for scanning. Finally, the tetrahedral torso with the command center and MTDN were examined.

### Fluorescence measurements

To optimize the reaction conditions, the concentration of the MTDN, reaction time, reaction temperature, and reaction pH were investigated. Sensitivity was measured using different concentrations of miR-21 (0.5 nM to 25 nM) incubated with MTDN. Specificity tests were conducted by adding miR-21, various other miRNAs, or a mixture of these miRNAs to the MTDN solution. The fluorescence spectra of the post-reaction sample under the above reaction conditions were captured from 500 nm to 600 nm upon excitation at 480 nm utilizing an F-7000 fluorescence spectrophotometer (Hitachi, Japan).

### Quantifying Dox in MTDN

To quantify Dox within MTDN, the fluorescence intensity at the peak was sequentially measured after diluting Dox solutions with TAMg buffer, achieving various final concentrations of Dox ( 0.1 μM to 10 μM). A linear relationship was established from these measurements. Subsequently, a 1 μM Dox solution (10 mM) was mixed with varying concentrations of MTDN from 1 nM to 100 nM at 37 °C for 12 h. Excess Dox was collected by centrifugation. The fluorescence intensity at the peaks of residual Dox was recorded. The amount of Dox encapsulated within the MTDN was quantified by subtracting the residual Dox concentration from the initial Dox concentration. The emission spectra were obtained upon excitation at 490 nm and a photomultiplier tube (PMT) voltage of 600 V (Hitachi Ltd., Japan).

### Cell culture

Complete medium was prepared using DMEM (Gibco, C11995500BT) or RPMI-1640 medium (Gibco, C11875500BT), supplemented with 10% fetal bovine serum (FBS, Gibco, A5669401) and 1% penicillin-streptomycin (Gibco, 15140122). HEK293T cells and MCF-7 cells (human breast cancer cell line) were cultured in the complete DMEM medium, and HC11 cells (murine mammary epithelial cells) and 4T1 cells (murine mammary carcinoma cells) were cultured in the complete RPMI-1640 medium in a humidified incubator at 37 °C 5% CO_2_.

### Cytotoxicity test

The cell suspension was collected and adjusted to a concentration of 5 × 10⁴ cells/mL. A 100 μL aliquot of the suspension was dispensed to each well of a 96-well plate. After removing the medium, the cells were incubated with complete DMEM containing various concentrations of MTDN (0, 25, 50, 100, 150, and 200 nM) to evaluate the cytotoxicity of MTDN. After 24 h, the biocompatibility of MTDN was assessed using the CCK-8 testing. Two cancer cell lines were treated with PBS, MTDN, UTDN-D, MTDN-D, and F-D, respectively. In the UTDN-D, MTDN-D, and F-D treatment groups, the equivalent concentration of Dox was 2 μM for MCF-7 cells and 4 μM for 4T1 cells. After 24 h, the proliferation inhibition was analyzed using the CCK-8 assay.

### RT-qPCR analysis

TRIzol reagent (Invitrogen, Carlsbad, CA, USA) was used to extract total RNA. For miR-21 and RNU6B, reverse transcription and qPCR were performed using the miRNA First Strand cDNA Synthesis (by Stem-loop) Kit and the 2 × SG Fast qPCR Master Mix Kit, respectively. The fold change in miR-21 expression between cancer cells (MCF-7 and 4T1 cells) and healthy cells (HEK293T and HC11 cells) was analyzed using the 2^ΔΔCt^ method after the expression of miR-21 was normalized against RNU6B using the 2^-ΔCt^ method.

### Western blot analysis

For the intrinsic apoptotic pathway assay, 4T1 cells were treated with PBS, MTDN, UTDN-D, MTDN-D, and F-D for 24 h. In the UTDN-D, MTDN-D, and F-D treatment groups, the equivalency concentration of Dox was 4 μM. The post-treatment cells were collected, and total proteins were extracted using lysis buffer supplemented with 1× PMSF. Protein concentration was quantified using the BCA protein assay kit. The proteins were then separated by SDS-PAGE and detected using electrochemiluminescence. The band intensity on the SDS-PAGE was measured using ImageJ software and analyzed with GraphPad Prism software.

### Cell uptake analysis

To analyze the endocytosis time, MCF-7 cells were incubated with Cy5-labeled MTDN (100 nM) for 0.5, 1, 2, 4, and 6 h. For endocytosis pathway analysis, MCF-7 cells were pre-treated with different endocytosis inhibitors: chlorpromazine (CPZ, 10 μM), amiloride (AMI, 1 mM), methyl-β-cyclodextrin (MCD, 3 mM), and genistein (GEN, 20 μM) for 1 h. After pre-treatment, Cy5-labeled MTDN was added and incubated with the cells for 6 h. Then, PBS was utilized to wash cells and remove uninternalized MTDN. Fluorescence signals were quantified using a BD FACSCanto™ II flow cytometer (BD Biosciences).

### Confocal laser scanning microscopy characterization

For live cell imaging, four cell lines (MCF-7, HEK293T, HC11, and 4T1 cells) were cultured on confocal glass dishes and treated with UTDN or MTDN for 4 h (functional H2 was labeled with FAM and BHQ2); the final concentration was 200 nM. The residual MTDN was removed by washing with PBS. Nuclei and mitochondria were then stained using Hoechst 33342 and MitoTracker Red CMXRos, respectively. For intracellular release and distribution analysis of Dox, cells were incubated with MTDN-D or F-D, followed by staining of mitochondria with MitoTracker Green and nuclei with Hoechst 33342, according to the manufacturer's instructions. All cellular fluorescence images were acquired using a Zeiss LSM780 confocal laser-scanning microscope (Zeiss, Germany).

### Apoptosis analysis

Suspensions of MCF-7 and 4T1 cells (5 × 10^5^ cells/well) were plated into 6-well plates. After cell adhesion, the cells were treated with PBS, MTDN, UTDN-D, MTDN-D, or F-D for 24 h. In the UTDN-D, MTDN-D, and F-D treatment groups, the equivalent concentration of Dox was 2 μM for MCF-7 cells and 4 μM for 4T1 cells. Post-treatment, cells were harvested and stained with Annexin V-FITC (5 μL) and propidium iodide (10 μL) in the dark for 15 min. The stained cells were analyzed using flow cytometry, and apoptosis data were processed using FlowJo software (version 10.6.2).

### Mitochondrial functions analysis

MCF-7 cells were treated with PBS, MTDN, UTDN-D, MTDN-D, or F-D for 24 h. In the UTDN-D, MTDN-D, and F-D treatment groups, the equivalent concentration of Dox was 2 μM. The mitochondrial morphology was characterized using biological transmission electron microscope (Bio-TEM) analysis. Mitochondrial membrane potential (MMP) was assessed using the JC-1 probe, which stains mitochondrial aggregates (red) and monomers (green). Cells were stained with JC-1 for 30 min at 37 °C, followed by three washes with a preheated culture medium. Intracellular reactive oxygen species (ROS) levels were evaluated using the DCFH-DA probe. The probe was diluted in an FBS-free culture medium at a 1:1000 ratio and incubated with treated cells for 20 min at 37 °C. The excess probe was removed by washing with the FBS-free culture medium. Confocal microscopy was employed to capture images of the stained cells, and the mean fluorescence intensity was quantified using ImageJ software.

### Orthotopic breast cancer model for diagnostic imaging and antitumor therapy

The 4T1 cells (2 × 10⁶ cells/100 μL) were transplanted into the mammary glands of 6-week-old female Balb/c mice to establish a primary tumor model mimicking human breast cancer. Body weight and tumor size were recorded every other day. Tumor volume was calculated using the formula: V = a × b²/2, where a is the maximum diameter and b is the minimum diameter. For diagnostic imaging, percutaneous intratumoral injection of MTDN (100 μL, 500 nM) labeled with Cy5.5 and BHQ2 on H2 was performed once the tumor volume reached approximately 100 mm³. An equal volume of MTDN was injected into the mammary glands of healthy mice as a control. Living images were acquired at 1, 2, 4, 8, 12, 24, 48, and 72 h post-injection using the IVIS Spectrum CT (PerkinElmer, USA), and epi-fluorescence in mice (n = 3) was quantified using Living Image software (version 4.4).

For oncotherapy, mice with orthotopic breast tumors (five mice per group) were intratumorally injected with PBS, MTDM, UTDM-D, MTDM-D, or F-D every two days (in UTDN-D, MTDN-D, F-D, the equivalent concentration of Dox was 2 mg/kg body weight). Two weeks post-injection, all mice were sacrificed. Vital organs (heart, liver, spleen, lung, and kidney), primary tumors, and blood samples were collected. Tissues were paraffin-embedded, sectioned, and subjected to hematoxylin and eosin (H&E) staining and immunohistochemical analysis. Blood samples were used for biochemical examinations. All animal experiments were conducted in strict compliance with the guidelines of the Institutional Animal Care and Use Committee of the Army Military Medical University (AMUWEC20225084).

### Statistical analysis

All data are presented as means ± SD. Differences between treatment groups were analyzed using either a Student's t-test or a one-way analysis of variance (ANOVA), as appropriate, with GraphPad Prism software (version 10.1.0). A P-value of less than 0.05 was considered statistically significant.

## Supplementary Material

Supplementary figures and table.

## Abbreviations

CHA: catalytic hairpin assembly
mitomiRs: Mitochondrial miRNAs
TPP: triphenylphosphine
Dox: doxorubicin
MTDN: mitochondria-targeting DNA nanorobots
UTDN: mitochondria-untargeted DNA nanorobots
F-D: free Dox
ROS: reactive oxygen species
tFNA: tetrahedral framework nucleic acid
ssDNA: single-strand DNA
LOD: the limit of detection
mtDNA: mitochondrial DNA
CCK-8: cell counting kit-8
AFM: atomic force microscopic
PMSF: Phenylmethanesulfonyl fluoride
CPZ: chlorpromazine
AMI: amiloride
MCD: methyl-β-cyclodextrin
GEN: genistein
MMP: Mitochondrial membrane potential
