# Supplementary material for: DNA nanorobot for mitochondria-targeted microRNAs detection and tailored regulation
Source: Theranostics. 2025 Mar 21;15(10):4638–53. doi: 10.7150/thno.105762 (PMC11984390; doi:10.7150/thno.105762)

DNA nanorobot for mitochondria-targeted microRNAs detection and tailored regulation

Ping Huang[1], Xiaoqi Tang[1], Shuang Zhao[1], Jie Luo[1], Yu Tang[1], Binpan Wang[1], Zuowei Xie[1], Xianlan Wu[1], Shuang Xie[1], Ming Chen[1,2], Kai Chang[1]**

[1]Department of Clinical Laboratory Medicine

Southwest Hospital

Third Military Medical University (Army Medical University)

30 Gaotanyan, Shapingba District, Chongqing 400038, P. R. China

[2]College of Pharmacy and Laboratory Medicine

Third Military Medical University (Army Medical University)

30 Gaotanyan, Shapingba District, Chongqing 400038, P. R. China

*Email: chming1971@126.com (M.C.) and changkai0203@tmmu.edu.cn (K.C.)

Table S1. Oligonucleotides sequences and modification

Names	Sequence (5'→3')
S1	CCAGGCAGTTGAGTCGAACATTCCTAAGTCTGAAATTTATCACCC GCCATAGTAGACGTATCA
S2	AGCAGCGGGTTGAAGTACTTTTCTTGCTACACGATCAGACTTAGG AATGTTCGATCATGCGAGGGTCCAATACCGTCGATTACAG
S3	TAATAAGGTGATAAAACGTGTAGCAAGCTGTAATCGACGGGAAGA GCATGCCCATCCACTACTATGGCG
S4	CGATATAATGACCCTGTGTTTTCCTCGCATGTCTCAACTGCCTGGT GATACGAGGATGGGCATGCTCTTCCCGACGGTATTGGAC
S3-TPP	TPP-TAATAAGGTGATAAAACGTGTAGCAAGCTGTAATCGACGGGA AGAGCATGCCCATCCACTACTATGGCG
S1-Cy5	CCAGGCAGTTGAGTCGAACATTCCTAAGTCTGAAATTTATCACCC GCCATAGTAGACGTATCATTTTGGT-Cy5
Hairpin 1 (H1)	GTACTTCAACCCGCTGCTTTTTTTTCAACATCAGTCTGATAAGCTA CCTCACACGAATTGTAGCTTATCAGACT
Hairpin 2 (H2)	CTCAGGGTCATTATATCGTTTTTTTAAGCTACAATTCGTGTGAGGT AGCTTATCAGACTCTCACACGAATT
H2-FAM-BHQ1	CTCAGGGTCATTATATCGTTTTTTTAAGCTACAATTCG/i6FAMdT/GT GAGGTAGCTTATCAGACTC/iBHQ1dT/CACACGAATT
H2-Cy5.5-BHQ2	CTCAGGGTCATTATATCGTTTTTTTAAGCTACAATTCG/iCY5.5dT/GT GAGGTAGCTTATCAGACTC/iBHQ2dT/CACACGAATT
miRNA-21(miR-21)	UAGCUUAUCAGACUGAUGUUGA
let-7	UGAGGUAGUAGGUUGUAUAGUU
has-miRNA-10b	UACCCUGUAGAACCGAAUUUGUG
has-miRNA-128	UCACAGUGAACCGGUCUCUUUC
has-miRNA-155	UUAAUGCUAAUCGUGAUAGGGGUU
has-miR-21-F	CGCGCGCGTAGCTTATCAGACTGA
has-miR-21-R	ATCCAGTGCAGGGTCCGAGG
has-miR-21-RT	CTCGTATCCAGTGCAGGGTCCGAGGTATTCGCACTGGATACGAG TCAACA
U6-F	AGAGAAGATTAGCATGGCCCCTG
U6-R	ATCCAGTGCAGGGTCCGAGG
U6-RT	GTCGTATCCAGTGCAGGGTCCGAGGTATTCGCACTGGATACGAC AAAATA

Figure S1. AFM images acquired after MTDN responded to the target miR-21 (MTDN: 200 nM, miR-21: 20 nM). Scale bar: 30 nm.

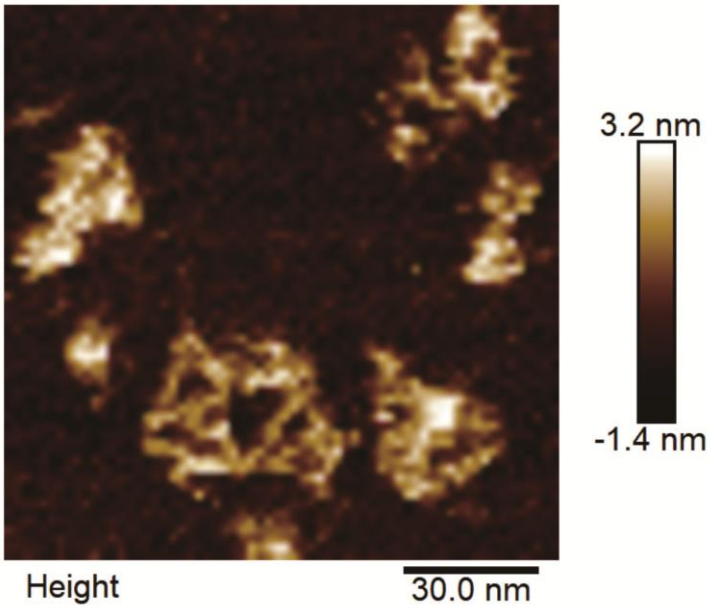

Figure S2. PAGE electrophoresis analysis on the feasibility of miR-21 detection using MTDN (MTDN: 200nM, miR-21: 100 nM).

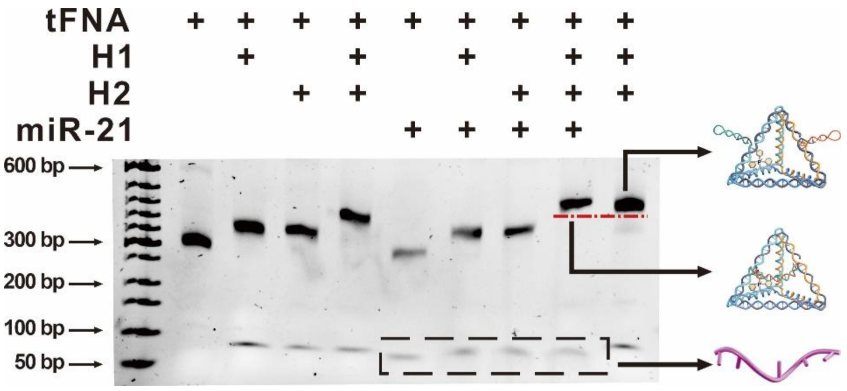

Figure S3. Structural stability of the MTDN and free functional hairpin in 10% fetal bovine serum, characterized by native PAGE electrophoresis.

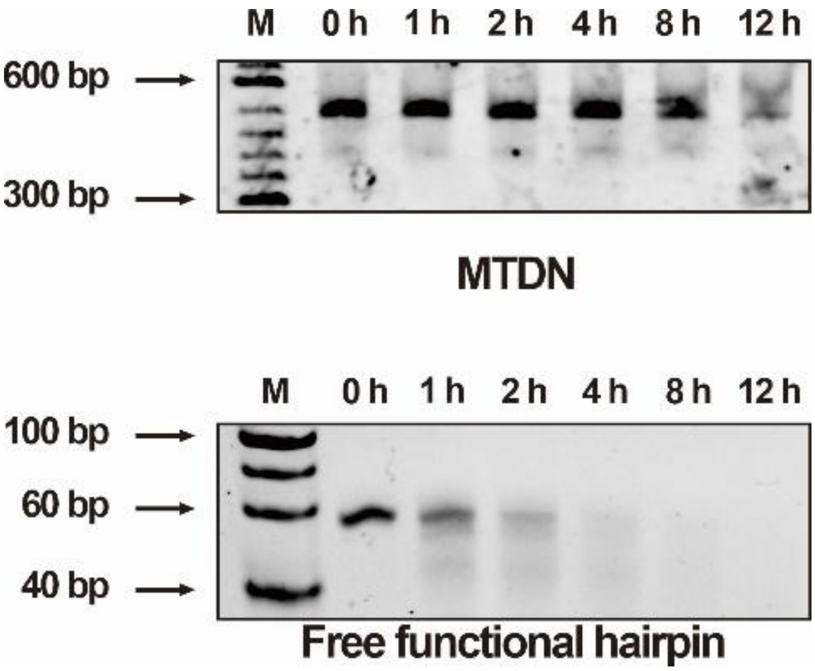

Figure S4. Images of isolated mitochondria from live cells captured using biological transmission electron microscopy. Scale bar: 2000 nm and 600nm.

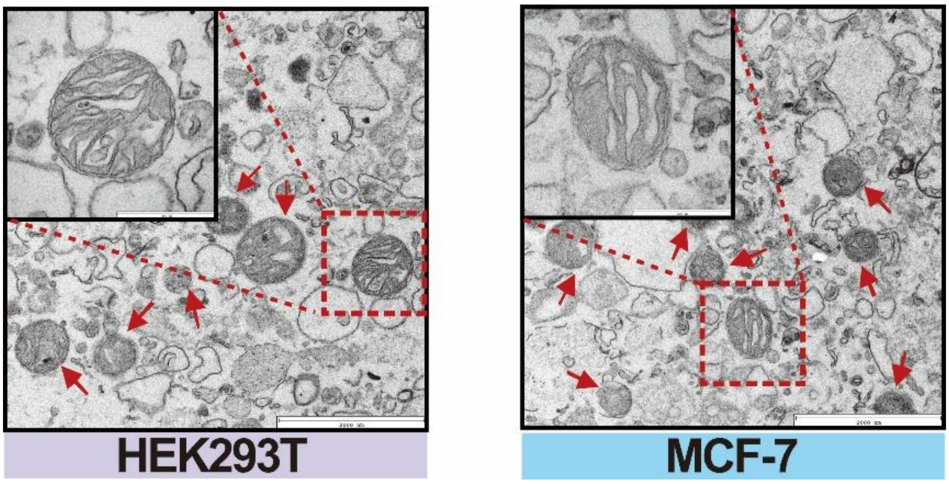

Figure S5. Confocal images of 4T1 and MCF-7 cells using UTDN to detect cellular miR-21. Scale bars: 20 μm. Overlap analysis indicates the degree of fluorescence colocalization of mitochondria (red) and miR-21 (green) along the white line using ImageJ software.

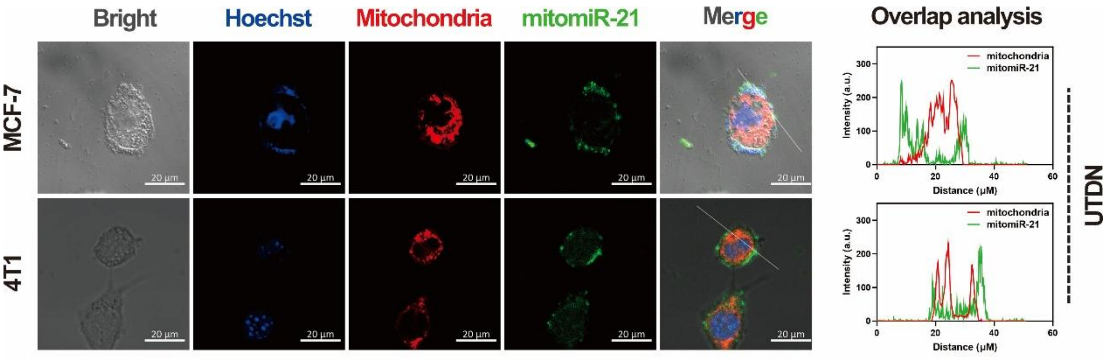

Figure S6. Fluorescence spectra of free Dox at different concentrations in the TAMg buffer (pH: 7.4).

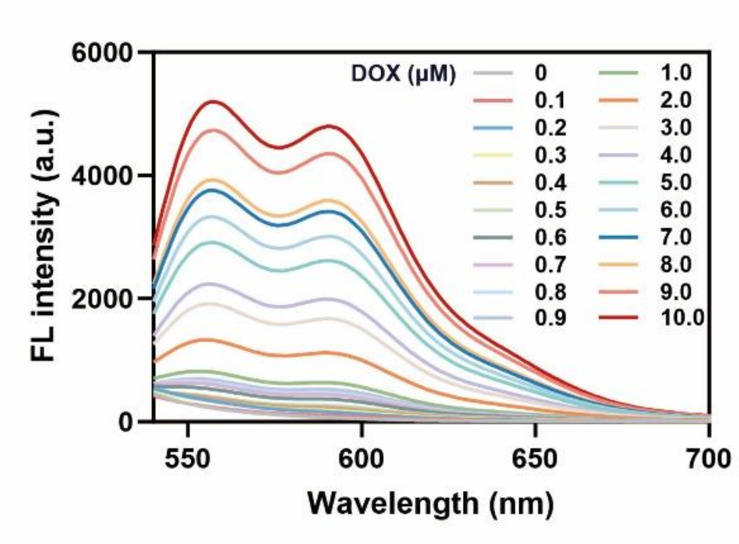

Figure S7. The process of MTDN-D and UTDN-D characterized by native.

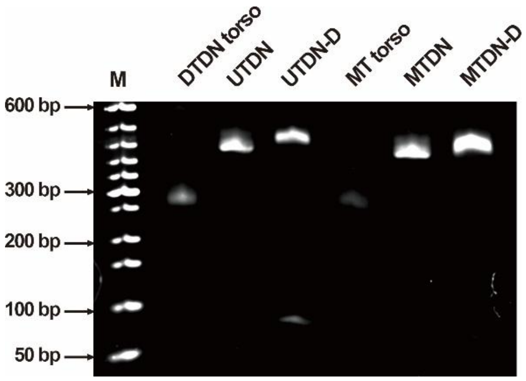

Figure S8. Cellular localization of Dox delivered by MTDN measuring by confocal microscopy. Scar bar: 20 nM.

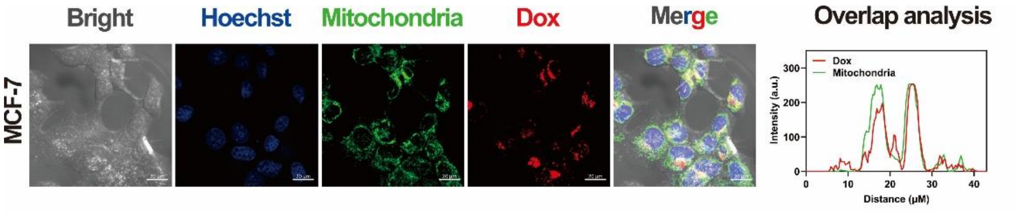

Figure S9. The mice' liver function tests (LFTs) using serum biochemical analysis after treatment with PBS, MTDN, UTDN-D, MTDN-D, F-D for two weeks.

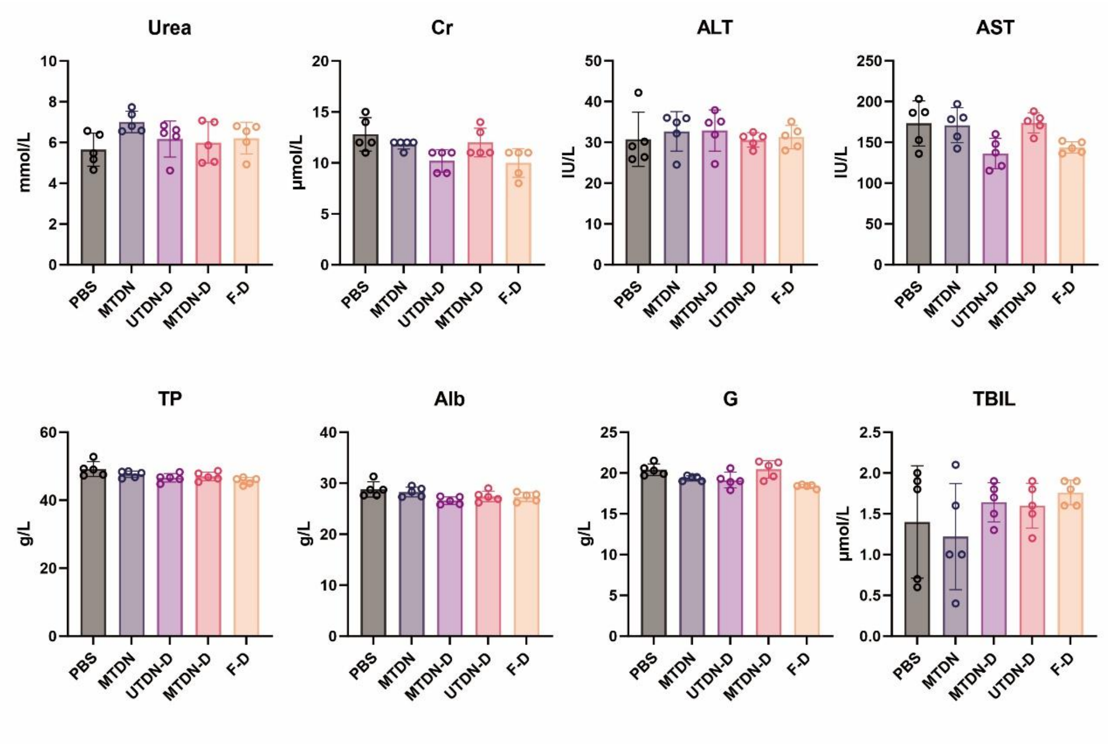

Figure S10. Analysis of primary structures in the major organs using H&E staining. Scar bar: 100 nM.

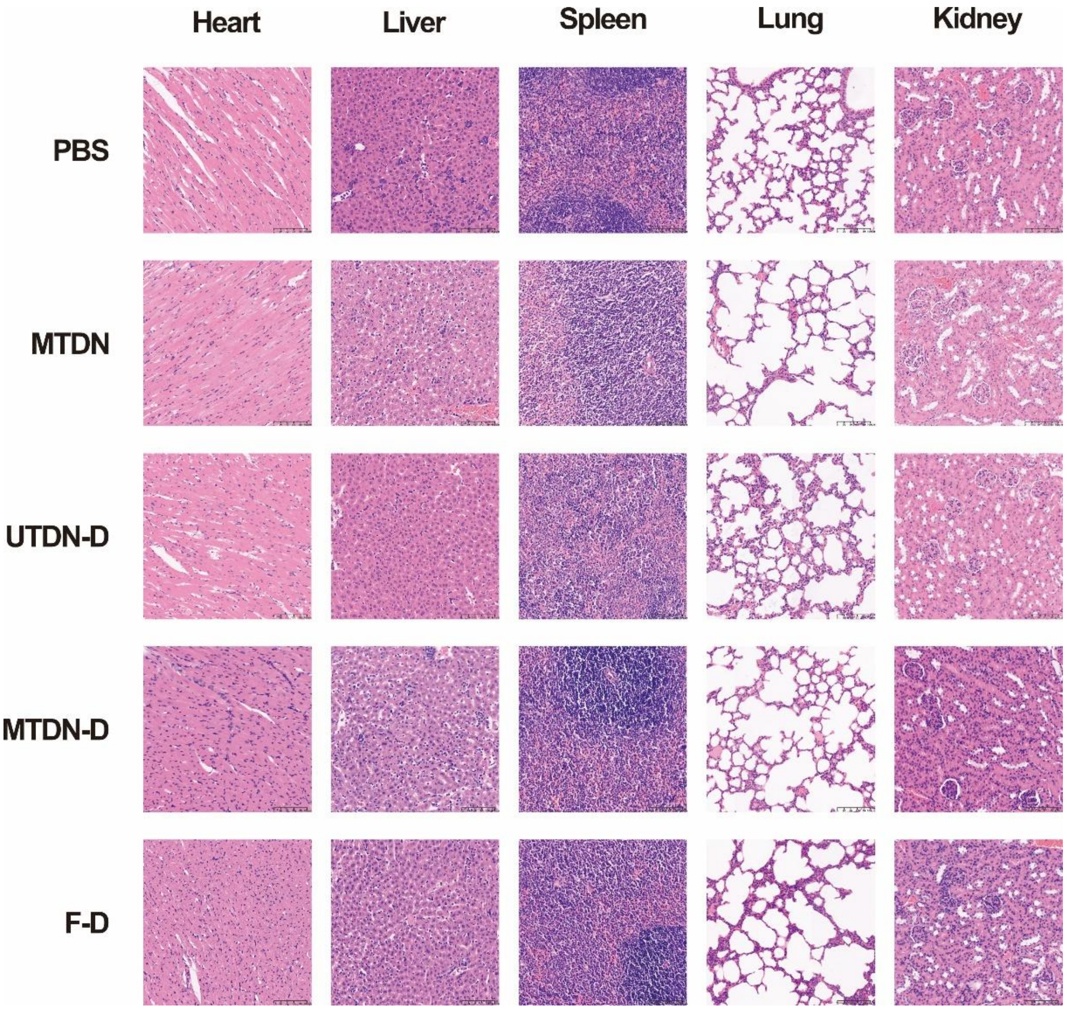

Supplement: Supplementary file 1 — Supplementary figures and table. [file thnov15p4638s1.pdf]
